# Making the connection: social networks and type 2 diabetes among Black/African American Men: mixed-methods study protocol

**DOI:** 10.3389/fpubh.2025.1483880

**Published:** 2025-05-23

**Authors:** Tyler Prochnow, Matthew Lee Smith, Megan S. Patterson, Jeong-Hui Park, Ledric D. Sherman

**Affiliations:** ^1^School of Public Health, Texas A&M Health Science Center, College Station, TX, United States; ^2^Center for Health Equity and Evaluation Research, Texas A&M University, College Station, TX, United States; ^3^Center for Community Health and Aging, Texas A&M University, College Station, TX, United States

**Keywords:** type 2 diabetes, men’s health, social networks, mixed-methods, self-management

## Abstract

This mixed-methods study protocol investigates the role of social networks in Type 2 diabetes (T2D) self-management among Black/African American (B/AA) men, a population disproportionately affected by T2D. The study employs a convergent design, combining quantitative social network analysis with longitudinal qualitative interviews. A nationally representative sample of 1,200 B/AA men with T2D will complete an online survey assessing their social networks, T2D self-management practices, and related psychosocial factors. A subset of 65 participants will engage in semi-structured interviews at two timepoints 6 months apart to explore the formation and evolution of supportive relationships. The study aims to: (1) identify specific aspects of social networks related to T2D self-management adherence, and (2) characterize the formation and evolution of relationships that improve T2D self-management strategies. Quantitative data will be analyzed using multivariate and multilevel regression techniques, while qualitative data will undergo thematic analysis. This comprehensive approach will provide insights into the structure and function of social networks among B/AA men with T2D, potentially informing culturally tailored interventions to improve T2D outcomes in this underserved population. The study’s innovative focus on the broader social context of T2D management among B/AA men has the potential to address health disparities and contribute to more effective strategies for reducing the burden of T2D in this population.

## 1 Introduction

Type 2 diabetes (T2D) represents a critical public health challenge, particularly among Black/African American (B/AA) males who are disproportionately affected by the disease. Approximately 13% of B/AA men are diagnosed with T2D compared to 8% of the general population in the United States (1). This disparity highlights the need for targeted research about T2D self-management within this demographic to prevent severe complications such as amputations, kidney failure, glaucoma, neuropathy, and stroke (1). Effective self-management is vital to manage and slow the progression of T2D; however, the long-term demands of managing T2D pose significant challenges, especially for B/AA males. Poor self-management has been associated with increased hospitalization and emergency department visits among B/AA older adults with diabetes (2). Research indicates that social networks and personal communities are critical in T2D self-management (3), yet their functioning and evolution among B/AA men with T2D remain poorly understood.

While previous studies have examined the influence of family members, spouses, and peers on T2D self-management (4, 5), there remains a gap in knowledge regarding the broader social networks of B/AA males with T2D. Understanding the formation, utilization, and evolution of these social networks is crucial for developing effective, culturally-appropriate interventions to improve T2D self-management among B/AA men (6). The socio-ecological model emphasizes the importance of considering multiple levels of influence on health behaviors, including interpersonal, community, and societal factors (7). In the context of T2D self-management among B/AA males, this approach suggests the need to examine individual behaviors and the social and cultural contexts that shape these behaviors. Research has shown that B/AA men face unique barriers to healthcare utilization and chronic disease management, including (but not limited to) cultural norms, masculine identity, and experiences of discrimination in healthcare settings (8, 9). Recent studies have highlighted the potential of social network interventions to improve health outcomes for various chronic conditions (10). However, there is a paucity of research specifically examining the role of social networks in T2D management among B/AA men (11). This gap is particularly concerning given the high prevalence of T2D within this population and the potential for social support to improve self-management behaviors (12).

The influence of masculinity on health behaviors and healthcare utilization among B/AA men has been increasingly recognized as a critical factor in T2D management (13, 14). Traditional masculine norms often conflict with health-promoting behaviors, posing significant barriers to effective T2D self-management (15). For instance, some B/AA men may perceive seeking medical help or adhering to a strict diet and exercise regimen as a sign of weakness or vulnerability, which can hinder their engagement with healthcare services and adherence to treatment plans (16). Furthermore, racial homophily in social networks—where individuals tend to associate with others who are racially similar (17)—and the unique characteristics of B/AA extended family, friendship, and congregational support networks can play a significant role in health behaviors (18). These networks often provide emotional support, shared understanding, and resources that can facilitate or impede diabetes management efforts (19). Consequently, culturally tailored approaches that leverage these social support systems are essential for improving T2D outcomes among B/AA men. Interventions designed to enhance social support from family, friends, and community organizations, while also addressing masculine norms, have shown promise to promote better health behaviors and outcomes (20, 21). Such approaches must consider the broader social determinants of health, including socioeconomic status and access to healthcare, to effectively address the disparities in T2D management within this population (22). These structural factors often create systemic barriers as well as alter the social support networks themselves which in turn limits the effectiveness of individual or network-level interventions alone.

The purpose of this protocol paper is to report our approach to investigate specific aspects of social networks related to T2D self-management among B/AA males. Once data are collected, the study will aim to address this knowledge gap by identifying the specific aspects of social networks related to T2D self-management among B/AA males. By employing a mixed-methods approach that combines quantitative social network analysis with qualitative interviews, we seek to identify key social predictors of T2D self-management quality and characterize how supportive relationships are established and maintained over time (23). This approach allows for a more comprehensive understanding of the complex interplay between social relationships and T2D management behaviors. The findings from this study will have the potential to inform the development of novel, culturally tailored interventions that leverage social support networks to improve T2D self-management among B/AA males. Such interventions could address not only individual health behaviors, but also the broader social context in which these behaviors occur, potentially leading to more sustainable improvements in T2D outcomes (24).

## 2 Methods

### 2.1 Study design and aims

This study protocol employs a convergent mixed-methods approach to examine the role of social networks in T2D self-management among B/AA males. The study design consists of two primary components: (1) a quantitative survey using social network analysis; and (2) longitudinal qualitative interviews. This approach allows for a comprehensive understanding of both the broad patterns and nuanced experiences of B/AA men managing T2D within their social contexts (25). The quantitative component will provide generalizable data about network structures and their associations with T2D management outcomes, while the qualitative component will offer rich, contextual insights into the formation, evolution, and utilization of these networks over time (11, 26). By integrating these methods, we aim to capture the complexity of social influences on T2D self-management, addressing a critical gap in the literature regarding the unique experiences of B/AA men (6).

The first aim of this study identifies specific aspects of B/AA men’s relationships (e.g., specific family members, friends, health care providers, or other meaningful individuals) that significantly influence T2D self-management adherence. This aim will be primarily addressed through the cross-sectional quantitative survey, which will employ validated measures of T2D self-management and a comprehensive assessment of participants’ social networks. By examining the characteristics of these networks and their associations with T2D management outcomes, we aim to uncover key network features that may support or hinder effective self-management (3, 12). We hypothesize that networks diverse in support (have different people supplying different types of support) will be associated with better T2D self-management adherence. This hypothesis is based on previous research suggesting that tightly knit, supportive networks can provide consistent encouragement and practical assistance for chronic disease management (27). Additionally, we expect that the presence of network members who also have T2D or who are health professionals will be positively associated with self-management adherence, as these individuals may provide more informed and relevant support (5).

The second aim is to characterize the formation and evolution of dyadic relationships that result in improved T2D self-maintenance strategy adherence among B/AA males. This aim will be addressed through the study’s quantitative and qualitative components. The longitudinal qualitative interviews will provide in-depth insights into how these relationships develop and change over time, while the cross-sectional quantitative measures will allow for statistical analysis of network composition and their associations with T2D management outcomes. By combining these approaches, we aim to develop a nuanced understanding of the dynamic nature of social support in T2D management among B/AA men, informing future interventions that can effectively leverage these social resources (6, 28). We hypothesize that participants will form and maintain relationships based on theoretical constructs of transitivity (friends of friends becoming friends), proximity (geographically close individuals forming connections), and comfort within “third places” or contexts that provide a comfortable space outside of work and home to socialize and connect. Furthermore, we predict that relationships that evolve to provide more diabetes-specific support over time will be associated with improved T2D self-management. This hypothesis is grounded in the network episode model, which suggests that people often turn to those in their networks for support during health challenges, but they may not limit discussions about important life and health matters solely to their closest support network (29, 30).

### 2.2 Conceptual framework

Our study is guided by an integrated theoretical framework that harmonizes both quantitative and qualitative approaches to understanding T2D self-management among B/AA men. This unified conceptual model positions social networks as the critical connecting mechanism between individual health behaviors and broader social contexts.

At the core of our framework, the Network Episode Model serves as the integrative theoretical foundation (29, 31). The Network Episode Model conceptualizes health management as a dynamic process in which individuals strategically mobilize different network resources during various health challenges (32). This framework directly corresponds to our quantitative measures of network composition (percentages of relationship types), structure (network size, relationship heterogeneity), and interaction patterns (communication frequency, diabetes-specific discussions). Through the NEM lens, we examine how these measurable network characteristics influence T2D management behaviors and outcomes, while our qualitative approach explores the meanings, experiences, and evolution of these network processes over time. Complementing the Network Episode Model, we incorporate House’s Social Support Theory to classify and measure specific support functions (33). Quantitatively, this allows us to assess the distribution and prevalence of emotional, instrumental, informational, and appraisal support within networks; qualitatively, it enables us to explore how these different forms of support are experienced, requested, and provided through interpersonal dynamics and communication patterns. Further, a Social Capital Framework unifies our approach by examining how social connections facilitate access to resources (34). Our quantitative measures distinguish between bonding and bridging capital through relationship type distributions and diversity indices, while our qualitative methods delve into how these connections are leveraged in daily diabetes management and how trust and reciprocity develop within these relationships. To ensure cultural relevance, we integrate the Health Disparities and Discrimination Framework and masculinity theory throughout both methodological approaches (13, 35). Quantitatively, we measure structural barriers and discrimination experiences using standardized instruments; qualitatively, we explore how these factors interact with cultural norms and gender expectations to shape network utilization and health behaviors.

This synergistic theoretical integration creates a comprehensive lens through which to examine T2D self-management among B/AA men. By applying these frameworks consistently across both quantitative and qualitative components, we establish conceptual coherence that allows findings from each method to inform and enhance the other. The quantitative analyses provide breadth and statistical validation of network patterns, while the qualitative exploration offers depth and context regarding how these networks function in real-world settings. Together, they generate a more complete understanding of the complex social dynamics influencing T2D management in this population than either approach could achieve independently.

### 2.3 Participants and procedures

Participants for this study will be a nationally representative sample of 1,200 B/AA males with T2D. Inclusion criteria are: (1) self-identification as B/AA; (2) male gender; (3) age 21 years or older; (4) self-reported T2D medical diagnosis; and (5) residence in the United States. The sample size was determined based on calculations to ensure adequate statistical power for planned analyses, considering the total population of B/AA men with T2D in the United States (approximately 3,036,917) and assuming a 50% rate of less-than-desirable social supports and networks for diabetes self-management (36, 37). Recruitment will be conducted through Cloud Research, which will allow for targeted selection of respondents who fit the specified criteria. This approach ensures that participants are anonymous to the research team and have already agreed to participate in survey research before starting the survey.

The study procedures involve two main components: a quantitative survey and longitudinal qualitative interviews. All 1,200 participants will complete the online quantitative survey, which will take approximately 20 min to complete. The survey will include validated measures of T2D self-management, social network characteristics, psychosocial factors, health behaviors, eHealth literacy, and health equity. Participants will be asked to provide information about their personal networks using a multiple name generator approach, following the Arizona Social Support Interview Schedule (38, 39). This will allow for a comprehensive assessment of participants’ social networks, including demographic characteristics of network members, the nature and quality of relationships, and the types of support provided.

For the qualitative portion, we aim to recruit 65 participants to engage in semi-structured interviews conducted at two time points 6 months apart. Each interview will last approximately 60 min and will be conducted virtually by the principal investigators. The interviews will focus on participants’ experiences with T2D self-management, the role of their social networks in this process, and how these relationships evolve over time. This longitudinal approach aligns with recommended diabetes care practices and allows for an in-depth exploration of the dynamic nature of social support in T2D management (40). To promote retention and minimize loss to follow-up, additional contact points will be established every 3 months between interviews, including email communications and phone calls. All study procedures have been reviewed and approved by the Institutional Review Board (#IRB2023-1311 M), and informed consent will be obtained from all participants prior to data collection.

### 2.4 Study variables

#### 2.4.1 Social network

The social network measure in this study utilizes a multiple name generator approach, following the Arizona Social Support Interview Schedule (38, 39). This comprehensive method allows for a detailed assessment of participants’ personal support networks (egocentric networks) related to their T2D management (41). Participants will be asked to list individuals in their life who: (1) give them advice; (2) they confide in; (3) provide practical support; and (4) make managing their T2D difficult, resulting in a comprehensive list of social network members. Participants can list the same person across multiple prompts when applicable. For each person nominated in their social network, participants are asked to specify their relationship type (spouse, child, parent, friend, sibling, extended family member, healthcare provider, coworker, roommate, neighbor, or other). This allows us to map the compositional diversity of support networks and identify which relationship categories were most prevalent. Participants also indicate whether each network member has T2D themselves (yes, no, I do not know), enabling analysis of disease homophily within networks and exploration of shared experiential knowledge. Health behaviors of network members are assessed through two key measures: perceived physical activity frequency and healthy eating habits, both rated on a four-point scale (never, rarely, sometimes, often). These measures provide insight into the health behavior modeling and social norms potentially occurring within participants’ social environments. The frequency of diabetes-specific discussions with each network member is measured on a similar four-point scale (never, rarely, sometimes, often), revealing communication patterns about T2D management. Perceived supportiveness specific to diabetes management is evaluated using a four-point scale (not at all supportive, a little supportive, sometimes supportive, very supportive), assessing the quality of support received. Finally, contact frequency with each network member is measured using a six-point scale ranging from several times daily to never (5 = several times daily, 4 = once daily, 3 = 3–5 days weekly, 2 = 1–2 days weekly, 1 = less than weekly, 0 = never), providing data on relationship intensity and accessibility of support.

Our analytical approach to social network data leverages a multifaceted calculation strategy to derive meaningful network metrics from the interpreter questions. Network composition variables are calculated as proportional measures, determining the percentage of each relationship type within participants’ networks. Specifically, we calculate the proportion of spouses, children, parents, friends, siblings, extended family members, and healthcare providers relative to total network size, providing insight into the relational composition of support systems. The percentage of network members with T2D is similarly calculated, enabling assessment of disease homophily within participants’ social environments. For network structure analysis, we determine network size by counting the total number of individuals named by each participant, while relationship heterogeneity is calculated using an entropy-based diversity index that quantifies the variety of relationship types present. Network interaction variables capture the dynamic aspects of these relationships; we calculate the percentage of contacts with whom participants communicate less than once weekly to assess interaction frequency distribution and assess the presence of weak ties. Mean communication frequency is computed by averaging the six-point scale responses across all network members, with higher values indicating more frequent contact throughout the network. Similarly, mean T2D communication frequency is derived by averaging the four-point scale responses regarding diabetes-specific discussions. Health behavior perceptions are quantified through mean perception scores for physical activity and healthy eating frequencies across all network members indicating social norms. Support quality is assessed both categorically, by calculating the percentage of network members described as “very supportive,” and continuously, through a mean social network support score averaged across all reported relationships. These calculated variables provide a robust framework for analyzing how network characteristics correlate with T2D self-management behaviors among B/AA men.

The use of this detailed social network measure provides a nuanced analysis of how different aspects of social networks relate to T2D self-management among B/AA males, providing insights into both the structure and function of these supportive relationships (42, 43).

#### 2.4.2 Self-care management for T2D

The Diabetes Care Profile (DCP) will comprise 16 profile scales encompassing patients’ diabetes control, attitudes towards diabetes, beliefs about diabetes, self-reported diabetes self-care practices, and challenges associated with diabetes self-care. The internal reliability of these profile scales, measured using Cronbach’s alpha, ranged from 0.60 to 0.95, demonstrating good to great reliability (44).

##### 2.4.2.1 Control T2D problems

The Control Problems Scale of the Diabetes Care Profile (DCP-CPS) survey instrument will be utilized to measure control issues related to T2D (45). First, the frequency of blood sugar monitoring among participants will be assessed using a single-item measure. Participants will be asked, “How frequently do you check your blood sugar levels?” Responses are recorded on a 5-point scale: 1 = Less than once a week, 2 = Weekly, 3 = 1–2 times a week, 4 = Daily, and 5 = More than once a day. Second, the scale includes four items to evaluate the frequency of specific diabetes-related events. Participants indicate the occurrence of particular symptoms and episodes over defined periods using a 6-point scale: 1 = 0 times, 2 = 1–3 times, 3 = 4–6 times, 4 = 7–12 times, 5 = More than 12 times, and 6 = Do not know. The items are: the frequency of low blood sugar reactions with symptoms (sweating, weakness, anxiety, trembling, hunger, headache) in the last month, severe low blood sugar reactions requiring assistance in the last year, high blood sugar symptoms (thirst, dry mouth and skin, increased sugar in urine, reduced appetite, nausea, fatigue) in the last month, and the presence of ketones in urine in the last month. Third, to assess the frequency of various diabetes management challenges experienced by participants, a scale consisting of 15 items will be used, each rated on a 5-point scale where 1 = never, 2 = rarely, 3 = sometimes, 4 = often, and 5 = very often. The specific items include the frequency of the following: being sick or having an infection, being upset or angry, taking the wrong amount of medicine, eating the wrong types of food, eating too much food, having less physical activity than usual, feeling stressed, eating too little food, having more physical activity than usual, and waiting too long to eat or skipping a meal. Possible scale scores for the frequency of diabetes management challenges range from 15 to 75, with higher scores indicating more frequent control problems related to T2D.

##### 2.4.2.2 Social and personal factors

The DCP—Social and Personal Factors Scale (DCP-SPFS) will be employed to assess the impact of diabetes on participants’ daily activities. First, this scale utilizes a 5-point rating system where 1 = Never, 2 = Rarely, 3 = Sometimes, 4 = Occasionally, and 5 = Often. Participants respond to the item: “How often has your diabetes kept you from doing your normal daily activities during the past year (e.g., could not go to work, work around the house, go to school, visit friends)?” Second, to measure participants’ perceptions of the impact of diabetes on their lives, the scale comprised 12 items rated on a 5-point Likert scale, where 1 = Strongly Disagree, 2 = Disagree, 3 = Neutral, 4 = Agree, and 5 = Strongly Agree. Specific items assessed included: “Paying for my diabetes treatment and supplies is a problem” and “Having diabetes makes my life difficult.” Possible scale scores for the impact of diabetes on daily activities range from 12 to 60, with higher scores indicating a greater perceived impact of diabetes on the participant’s life.

##### 2.4.2.3 Long-term care benefits

For this study, the DCP—Long-Term Care Benefits Scale (DCP-LTCBS) will be employed to evaluate participants’ beliefs regarding the benefits of optimal diabetes care. This scale utilizes a 5-point Likert scale, where 1 = Strongly Disagree, 2 = Disagree, 3 = Neutral, 4 = Agree, and 5 = Strongly Agree. Participants respond to items such as: “Taking the best possible care of diabetes will delay or prevent: eye problems,” “Taking the best possible care of diabetes will delay or prevent: kidney problems,” “Taking the best possible care of diabetes will delay or prevent: foot problems,” “Taking the best possible care of diabetes will delay or prevent: hardening of the arteries,” and “Taking the best possible care of diabetes will delay or prevent: heart disease.” The responses to these items will be aggregated to compute a composite score representing each participant’s beliefs in the long-term benefits of T2D care, with possible scores ranging from 5 to 25.

##### 2.4.2.4 Monitoring practices for T2D

The DCP—Monitoring Barriers and Understanding Management Practice Scales (DCP-MBUMPS) will be utilized to evaluate barriers to T2D monitoring and the frequency of management practices among participants (45). First, the scale includes two items related to T2D monitoring using urine and blood tests (e.g., “How many days a week have you been told to test urine sugar?” and “How many days a week have you been told to test blood sugar?”), with participants indicating the number of days per week they were advised to perform these tests. Second, the scale assesses the frequency of failed blood sugar tests due to various reasons through 10 items. Participants indicate how often they failed to test their blood sugar as instructed due to reasons such as forgetting, doubting the utility of testing, inappropriate timing or location, disliking the task, running out of test materials, cost, inconvenience, difficulty reading test results, inability to perform the test independently, infrequent changes in levels, and discomfort from finger pricks. Responses will be recorded on a 5-point Likert scale: 1 = rarely, 2 = sometimes, and 3 = often. The possible scores ranged from 10 to 30. Third, participants were provided with a list of seven educational programs and asked to select all that applied to them. The options include one-on-one counseling with a diabetes educator, nurse, or dietitian; group diabetes management classes; diabetes self-management education programs; nutrition classes specifically for managing diabetes; foot care workshops for preventing diabetes complications; other educational programs focused on living with diabetes (with an option to specify); and an option for not having received any formal diabetes education. Participants could select multiple programs or activities, and their responses were coded dichotomously (0 = not selected, 1 = selected). Lastly, the participants will respond to 10 items related to T2D monitoring barriers and understanding management practices. The scale assesses participants’ understanding of various aspects of diabetes care, including diet and blood sugar control, weight management, the role of exercise, use of insulin/pills, sugar testing, foot care, prevention of long-term complications, eye care, medications, and alcohol use and diabetes. Respondents rated their understanding of each item on a 5-point Likert scale. Possible scale scores for barriers to blood sugar testing range from 10 to 30, with higher scores indicating more frequent barriers to testing blood sugar as instructed.

##### 2.4.2.5 Self-care management for T2D

To examine the self-care management for T2D, the study will utilize the Summary of Diabetes Self-Care Activities (SDSCA) questionnaire (46). This instrument is a widely used tool for assessing diabetes self-care practices over a recent timeframe, either the previous week or month. The SDSCA evaluates key domains of self-care, including dietary habits, glucose monitoring, foot care, and adherence to self-care guidelines, by measuring the frequency or consistency of engagement in these activities (46). Participants report the frequency of each self-care activity over the past 7 days using an 8-point Likert scale (ranging from 0 to 7 days). Possible scale scores range from 0 to 7 for each self-care activity, with higher scores indicating more frequent engagement in the specific self-care activity over the past week.

##### 2.4.2.6 Self-regulatory efficacy for T2D

The Self-regulatory Efficacy for T2D will be measured using the Self-Efficacy for Diabetes (SED) Scale, a widely recognized instrument for assessing diabetes-specific self-efficacy (47). Developed and validated for the Diabetes Self-Management study, this 8-item scale employs a 10-point rating system (47). Participants’ responses to the items will be combined to calculate a composite score, representing each participant’s total self-regulatory efficacy for T2D, with possible scores ranging from 8 to 80. Lower scores indicated reduced self-efficacy, while higher scores reflected increased self-efficacy. The SED scale has demonstrated strong reliability, with excellent internal consistency (Cronbach’s α = 0.85) and test–retest reliability (intraclass correlation coefficient = 0.80) (48). Furthermore, the scale showed convergent validity, with item-scale correlations exceeding 0.50 (48).

#### 2.4.3 Mental health

##### 2.4.3.1 Stress, anxiety, and depression

The Depression, Anxiety, and Stress Scales (DASS-21) will be used to evaluate participants’ mental health status (49). Each item is rated on a 4-point Likert scale ranging from 0 to 3, reflecting the presence and severity of symptoms over the past week. Each subscale contains seven items, yielding total scores that range from 0 to 21, with higher scores indicating greater symptom severity. The DASS-21 has demonstrated strong reliability, with Cronbach’s alpha values of 0.81 for depression, 0.89 for anxiety, and 0.78 for stress (50). Additionally, the scale shows excellent internal consistency and robust discriminative, concurrent, and convergent validity (50).

##### 2.4.3.2 Loneliness

Participants’ loneliness will be measured using the UCLA 3-item Loneliness Scale. Initially developed as a 20-item questionnaire (51), the scale was condensed to three items in 2004 to facilitate its use in larger surveys and telephone interviews (52). The 3-item version has demonstrated reliability and validity, with a Cronbach’s alpha of 0.72 and a high correlation of 0.82 with the original UCLA Loneliness Scale (52). Participants will respond to the following items: “How often do you feel that you lack companionship?,” “How often do you feel left out?,” and “How often do you feel isolated from others?.” Responses were rated on a 3-point Likert scale: 1 = Hardly Ever, 2 = Some of the Time, and 3 = Often. Possible scale scores range from 3 to 9, with higher scores indicating more feelings of loneliness.

#### 2.4.4 Health behaviors

##### 2.4.4.1 Alcohol consumption

The Alcohol Use Disorders Identification Test-Concise (AUDIT-C) will be employed to investigate participants’ alcohol consumption (53). This concise screening instrument was designed to effectively identify individuals who are hazardous drinkers or have active alcohol use disorders, including alcohol abuse or dependence (53). The modified version includes three questions, each scored on a scale of 0 to 4 points, with a total possible score ranging from 0 to 12 (54). Higher scores generally indicate a greater likelihood that an individual’s alcohol consumption was adversely affecting their health. Alcohol consumption will be assessed using the following questions: “Within the past year, how often did you have a drink of alcohol?” (Responses coded as 0 = Never, 1 = Monthly, 2 = 2–4 times a month, 3 = 2–3 times a week, and 4 = 4 or more times a week), “Within the past year, how many standard drinks containing alcohol did you have on a typical day?” (Responses coded as 0 = 1 or 2, 1 = 3 or 4, 2 = 5 or 6, 3 = 7 to 9, and 4 = 10 or more), and “Within the past year, how often did you have six or more drinks on one occasion?” (Responses coded as 0 = Never, 1 = Less than monthly, 2 = Monthly, 3 = Weekly, and 4 = Daily or almost daily) (54). The internal reliability of the AUDIT-C has been reported as good, with a Cronbach’s alpha of 0.75 (55).

##### 2.4.4.2 Smoking habits

The Summary of Diabetes Self-Care Activities (SDSCA) questionnaire will be used to evaluate participants’ smoking habits (46). This questionnaire assesses key domains of self-care, including dietary and smoking habits, glucose monitoring, and adherence to self-care guidelines, by evaluating the absolute frequency or consistency of engagement in these activities (46). The SDSCA has demonstrated adequate reliability across various cultural backgrounds, with Cronbach’s alpha values exceeding 0.50 (56–58). Smoking habits were assessed with the question: “Have you smoked a cigarette—even one puff—during the past 7 days?” Responses were recorded on a 2-point Likert scale (1 = No, 2 = Yes). Additionally, participants will provide a free-response answer to the question, “How many cigarettes did you smoke on an average day?” which was treated as a continuous variable.

##### 2.4.4.3 Physical activity

Physical activity will be assessed using the International Physical Activity Questionnaire-Short Form (IPAQ-SF). The IPAQ-SF records activity across four intensity levels: 1) vigorous-intensity PA such as aerobics, 2) moderate-intensity PA such as leisure cycling, 3) walking, and 4) sitting. Additionally, participants were asked to respond to open-ended questions regarding the frequency and duration of their PA, specifically indicating the number of days per week and the number of minutes per day they engaged in PA. The original authors recommend the “last 7-day recall” version of the IPAQ-SF for PA surveillance studies due to its minimal reporting burden on participants. Test–retest reliability indicated good stability and high reliability (Cronbach’s α < 0.80) (59).

#### 2.4.5 Digital health

The eHealth Literacy Scale (eHEALS) is an 8-item tool designed to evaluate eHealth literacy by measuring consumers’ knowledge, comfort, and perceived skills in locating, assessing, and utilizing electronic health information for health-related decisions. Participants will respond on a 5-point Likert scale: 1 = Strongly Disagree, 2 = Disagree, 3 = Undecided, 4 = Agree, and 5 = Strongly Agree. Additionally, two supplementary items were recommended for use with the eHEALS to understand consumers’ interest in using eHealth: (1) “How useful do you feel the Internet is in helping you make decisions about your health?” (Responses were coded as 1 = Not useful at all, 2 = Not useful, 3 = Unsure, 4 = Useful, and 5 = Very Useful) and (2) “How important is it for you to be able to access health resources on the Internet?” (Responses were coded as 1 = Not important at all, 2 = Not important, 3 = Unsure, 4 = Important, and 5 = Very Important). Item analysis conducted at baseline yielded a highly reliable scale, with a Cronbach’s alpha of 0.88 (60). Possible scale scores range from 10 to 50, with higher scores indicating greater eHealth literacy.

#### 2.4.6 Health equity

The Expanded Everyday Discrimination Scale (EDS) aims to measure the frequency and impact of daily discriminatory experiences encountered by racial and ethnic minority groups (61). This tool consisted of 10 items that capture respondents’ experiences of mistreatment (61). Prior research has validated the Expanded EDS, showing strong psychometric properties, including high reliability and construct validity, with Cronbach’s alpha ranging from 0.80 to 0.88 (62).

#### 2.4.7 Sociodemographic

The study will collect a range of sociodemographic and anthropometric data, which include variables such as age, sex, race/ethnicity, rurality, educational levels, marital status, job status, annual household income, and Body Mass Index (BMI). Participants were defined based on specific criteria: age (21 years or older), sex (restricted to men), and race/ethnicity (limited to B/AA individuals). Rurality will be categorized into rural, suburban, urban, and other areas. Educational levels will be grouped into less than high school graduate, some college/2-year degree/no degree, and 4-year degree or higher. Marital status classifications include married/partnered, never married, divorced/separated, and widowed. Job status will be identified as student, employed, unemployed, retired, or unable to work. Annual household income will be reported in $25,000 USD increments. BMI will be calculated by dividing the participant’s weight (kg) by the square of their height (m^2^).

### 2.5 Qualitative inquiry

The qualitative data analysis will employ a phenomenological approach, which aims to uncover the essence of participants’ lived experiences with T2D self-management and their social networks (23). Analysis will begin with a thorough reading of all interview transcripts to gain a holistic understanding of the data. Following this, the research team will engage in a systematic coding process using MAXQDA qualitative data analysis software.

Initial coding will involve four major frameworks that coincide with the quantitative constructs collected. First, we will utilize House’s Social Support Theory Framework (33), which categorizes support into emotional, instrumental, informational, and appraisal dimensions, helping us identify how different types of social support influence T2D self-management behaviors among B/AA men. Second, Pescosolido’s Network Episode Model will frame our understanding of how individuals activate and navigate their social networks during health challenges, particularly examining network activation, navigation, content, and timing in relation to diabetes management decisions (29, 31). Third, the Social Capital Framework (34, 63) will illuminate how bonding (close-knit) and bridging (cross-group) social connections provide different resources and opportunities for diabetes care, with special attention to trust, reciprocity, and community norms. Fourth, the Health Disparities and Discrimination Framework, informed by Critical Race Theory (64), will guide our analysis of structural barriers, discrimination experiences, cultural factors, and resilience strategies that uniquely shape T2D management among B/AA men. Additionally, we will code for supplementary concepts including negative/non-supportive ties, family traditions, social norms around health behaviors, and individual barriers to diabetes management, providing a comprehensive understanding of the social dynamics influencing T2D self-care in this population. These coding frameworks are directly.

Particular attention will be paid to how participants described the formation and evolution of their social networks, the types of support they received, and how these networks influenced their T2D self-management practices. The analysis will also focus on identifying changes in these aspects over the two time points of data collection. To ensure rigor and trustworthiness, we will employ member checking, where preliminary findings are shared with a subset of participants for validation, and peer debriefing, where researchers not directly involved in the coding process will review and provide feedback on the analysis (65). The final step will involve synthesizing the findings into a coherent narrative that captures the essence of B/AA men’s experiences with social networks and T2D self-management, which will then be integrated with the quantitative findings to provide a comprehensive understanding of the phenomenon under study.

### 2.6 Analysis plan

The quantitative data analysis will employ a multi-faceted approach to examine the relationship between social network characteristics and T2D self-management outcomes among B/AA males. Initially, descriptive statistics will be used to characterize the sample and summarize key variables. To address our first aim, we will use Multivariate Analysis of Covariance (MANCOVA) and Generalized Estimating Equations (GEE) to determine significant differences in network-level variables between those indicating successful T2D management and those who do not, controlling for demographic variables. Network-level variables will include measures such as network size, density, diversity of support, and proportion of network members with health-related expertise. For more granular analysis, we will employ multi-level regression analysis using GEE to examine specific dyadic-level factors that promote T2D self-management adherence. This approach allows us to treat each alter (network member) as a unique data point while accounting for the nested structure of the data. Social network analysis techniques will be used to examine network structure (e.g., centrality measures) and composition. To address our second aim and capture changes over time, we will use repeated-measures linear mixed models to compare network characteristics and T2D management outcomes at baseline and follow-up time points. All analyses will be conducted using appropriate statistical software (e.g., SAS and R), with a significance level set at *p* < 0.05. To ensure the robustness of our findings, we will conduct sensitivity analyses to assess the impact of missing data and potential confounding variables.

### 2.7 Triangulation of mixed-methods

The integration of quantitative and qualitative data in this study will be accomplished through methodological triangulation, enhancing validity and comprehensiveness by examining social networks and T2D self-management through similar constructs but in different methods (25). The integration will be grounded in a unity of theoretical constructs used in both quantitative and qualitative forms (25). These theoretical constructs include the dimensions of social support, social norms, network activation, and cultural expectations. As a convergent design, results will be presented through joint displays of quantitative results alongside qualitative themes, allowing for direct comparison and identification of convergence or divergence (25). This continuous comparison will also enable us to pursue findings from one data set across to the other, creating a comprehensive narrative that captures both broad patterns of association between network characteristics and T2D outcomes and the rich contextual processes underlying these relationships, ultimately informing more effective, culturally tailored interventions for B/AA men with T2D.

## 3 Discussion

This study will address a critical gap in the literature by examining the role of social networks in T2D self-management among B/AA males, a population that bears a disproportionate burden of T2D and its complications. The significance of this research lies in its potential to illuminate the complex social dynamics that influence T2D management among this underserved group. By focusing specifically on B/AA men, we acknowledge the unique cultural, social, and health-related challenges they face, which are often overlooked in broader diabetes research (11). The findings from this study may inform more culturally tailored and effective interventions for T2D management, ultimately reducing health disparities in this population.

The innovation of this study lies in its comprehensive, mixed-methods approach to understanding social networks and T2D self-management. While previous research has examined the influence of family members and peers on diabetes management (4, 5), our study will take a broader view by considering the entire social network of B/AA men with T2D. The use of social network analysis combined with in-depth qualitative interviews will allow for a nuanced understanding about the structure and function of these networks. Furthermore, the longitudinal design of the qualitative component is innovative in its ability to capture the dynamic nature of social networks and their influence on T2D management over time, providing insights that cross-sectional studies cannot offer.

Results from this study may reveal specific characteristics of social networks that are associated with better T2D self-management among B/AA men. For instance, we may find that networks with a higher proportion of individuals who also have T2D or who work in healthcare are associated with better glycemic control and adherence to self-management behaviors. The qualitative findings may illuminate the processes through which B/AA men form and maintain supportive relationships for T2D management, potentially highlighting the importance of community spaces or shared experiences in fostering these connections. We may also uncover unique challenges that B/AA men face in leveraging their social networks for T2D management, such as cultural norms around masculinity or experiences of racial discrimination in healthcare settings.

The implications of these findings could be far-reaching. At the clinical level, healthcare providers could use this information to better assess and leverage the social resources of their B/AA male patients with T2D. For instance, they might incorporate questions about social network composition and support into their assessments or encourage patients to involve supportive network members in their care. At the community level, these findings could inform the development of peer support programs or community-based interventions that are tailored to the specific needs and social contexts of B/AA men with T2D. From a policy perspective, this research could highlight the need for broader social and community-level interventions to support T2D management, moving beyond individual-focused approaches. Ultimately, by enhancing our understanding about how social networks influence T2D self-management among B/AA men, this study has the potential to contribute to more effective, culturally appropriate strategies for reducing the burden of T2D in this population.
